# Habits of California Ostriches

**Published:** 1883-07

**Authors:** 


### HABITS OF CALIFORNIA OSTRICHES.

The editor of the Anaheim (Cal.) *Gazette* has been viewing the ostriches on a ranch near Cost Station. He says: "The female lays an egg on alternate days to the number of fifteen, when, if permitted to set, she considers her work done. If, however, her eggs are taken from her, she will lay thirty before she discovers the deception. And such eggs! The one showed us weighs three and a half pounds, and contains food sufficient to furnish a plentiful breakfast for four men. One would suppose that the flavor of such eggs would be unpleasantly pronounced. Such is not the case, however, the flavor not being as decided as that of duck eggs. What schoolboy has not read of the ostrich egg, and of its being hatched in the hot sun of Africa's sunny shore? But this pretty little legend, like many other cherished stories of the past, is all gammon. The chicks are brought forth in the good old way. The female sits on the eggs in the day time, and the male assumes that duty at night, allowing the female to seek rest and recreation while he attends to the household duties. It must be noted here that the male is much more solicitous for his household than is the female. It not unfrequently happens that the latter prefers to gad about rather than take her turn at setting, and on such occasions her lord and master administers to her a deserved chastisement by kicking her heartily around the paddock, until she manifests proper contrition, and signifies her willingness to settle down on the eggs. There is a moral somewhere about this incident which, when found, make a note of."

---

THE weaks sinews become strong by their conflict with difficulties. Hope is born in the long night of watching and tears.—*Dr. Chapin.*

THERE are words which can separate hearts sooner than sharp swords; there are words whose sting can remain in the heart through a whole life.

IF we keep well and cheerful and the mind constantly active, we never grow old. By and by we get to the end of the journey, but we never grow old.—*E. N. Kirk.*

BEWARE of what you say of others, because you only reveal yourself thereby. A man doesn't think to look behind the door unless he has sometimes stood there himself.

ORCHARDISTS in California are talking of cultivating squirrels to kill off the sparrows by sucking their eggs. When the job is finished cats will be employed to kill off the squirrels, and small boys will kill the cats. If cigarettes don't kill the small boys high living may.

